# “Forbidden Fish”: Did King Henry I Die of Lamprey Poisoning?

**DOI:** 10.7759/cureus.39298

**Published:** 2023-05-21

**Authors:** Matthew D Turner

**Affiliations:** 1 Transitional Year, Madigan Army Medical Center, Lakewood, USA

**Keywords:** food poisoning, king, england, listeria monocytogenes, poisoning, lamprey, medical history, henry i

## Abstract

For centuries, the sudden and mysterious death of King Henry I has been attributed to a large meal of lampreys that accidentally poisoned the unfortunate monarch. In this article, we conclude that lampreys were likely not the cause of the king’s illness, nor is it likely that he was deliberately poisoned. Although a wide variety of abdominal pathologies could have been responsible, we suggest that a sporadic central nervous system (CNS) infection of *Listeria monocytogenes *appears to be the most likely cause of Henry’s death, correlating with both his symptoms and rapid decline.

## Introduction and background

On the afternoon of November 25th, 1135, King Henry I of England returned from a long day of hunting in the forest of Lyons. The king loved hunting and was such a skilled tracker that he was nicknamed “Stag’s foot” [1]. The natural world had become a national priority for England under his rule, as English forests under his reign grew nearly to their maximum extent -- he even went so far to order those “who had land near the bounds of the forest [to] mutilate their dogs so that they could not hunt the deer” [1]. The 67- or 68-year-old king still displayed much of the “tremendous energy” of his youth [1] and returned to his lodge at Lyons-la-Forêt [2]. He appears to have been feeling well at this time, for he ordered his huntsmen to prepare for another hunt the next day [2]. Once within the small castle -- only recently constructed [3] to secure England’s restless Norman possessions and dangerous southern border with France [1] -- the king sat down for dinner. According to the chronicler Henry of Huntingdon, the king dined on a large dish of lampreys, against the advice of his physicians [1]. He fell ill during the night, quickly developing a chill, convulsions, fever [1], and “heavy sweating” [2]. The suddenness of the healthy king’s illness was utterly unexpected, and over the next several days his condition continued to deteriorate [2]. Soon, even Henry was aware that the end was near. He performed the last rites with Archbishop Hugh of the nearby settlement of Rouen, and conducted the last affairs of his reign -- freeing prisoners, allowing exiles to return, and determining the location of his resting place [1]. Of note, his nobles made conflicting claims about the king’s statement on the royal succession, with some swearing that he had left the throne to his daughter Matilda, while others claimed that it was meant to pass to his nephew Stephen -- if these conflicting tales are true, they paint a picture of a once-forceful and assertive monarch acting in a confusing and contradictory manner on his deathbed [2]. Henry’s illness progressed so rapidly that “the leading contenders in the succession were dispersed, and uncertainty about the future only served to heighten the tension of an interregnum” [2]. His daughter Matilda may have had enough time to rush to his side and secure her inheritance, but the father and daughter had been feuding, and she maintained her distance. By the time she realized her mistake, it was already too late [4]. Henry died on December 1 before the matter could be conclusively determined, thus ushering in the bloody period of civil war that would come to be known as The Anarchy [2]. While his entrails were buried at Notre-Dame-du-Pré, his body would not be buried at Reading Abbey until January 4, 1136. Henry of Huntingdon reported that Henry’s corpse had such a “strong, prevalent stench” that it killed the man who had been hired to remove the king’s brain. The chronicler grimly noted that the unfortunate man “was the last of many whom King Henry had put to death” [2].

Henry’s death would lead to far more widespread chaos. Unrest quickly broke out in Normandy and England proper as Stephen and Matilda jockeyed for power and the prospect of civil war loomed on the horizon [4]. In this tense environment, Henry of Huntingdon’s description of Henry I’s death quickly became the dominant story: “the king violated his doctor’s orders and dined on forbidden fish” [4]. Even in the modern day, “the unfortunate lamprey, combined with the monarch's greed, has been blamed for the death of this cantankerous king” [5]. In this article, we investigate if this etiology adequately explains the rapid progression of the king’s disease.

## Review

Death via lamprey?

Lampreys were a popular delicacy among the wealthy during the medieval period. Considered a delicacy, “nothing in the fish category was more prestigious in the Middle Ages than lamprey” [6]. For religious purposes, they were considered a fish, but in both taste and texture they are closer to “beef short-ribs or venison” [6], making them a convenient dish during periods of Catholic fasting [6]. English kings had a particular fondness for lampreys [7]; for centuries the city of Gloucester would present a lamprey pie annually at Christmas to the English crown [8]. The English kings consumed both sea lamprey from the English Channel and European river lamprey (*Lampetra fluviatilis*) [7]. The lampreys that Henry consumed on November 25, 1135 were likely *L. fluviatilis* [8].

Wild lamprey must be caught just prior to spawning, with a brief season of February to April, depending on the exact latitude [9], making it unlikely that Henry’s November meal came from the nearby Seine River. While nobility and royalty often bred lamprey in their own fishponds [10], it is doubtful that Lyons-la-Forêt had its own fishpond. Henry was visiting the area at the time to strengthen the southern border against Norman rebels and French invaders [4]. It is unlikely that the newly built fortress would have contained such luxuries. Recent excavations of the site have yielded no evidence of a fishpond [3]. Further complicating the issue is that, while “medieval preparations for lamprey were varied… all required killing it at the last moment and using its blood for the sauce” [6]. Freedman concludes that “the lamprey that supposedly dispatched” Henry likely originated from Nantes, “the most famous center for acquiring lamprey” in the Middle Ages [6]. The nearly 400-km distance from Nantes to Lyons-la-Forêt raises significant questions about the quality of the lamprey that Henry ate on November 25th. However, King John of England (1166-1216) and King Henry V (1387-1422) likewise ordered significant amounts of lamprey from Nantes while they were in Normandy, indicating that the quality of the fish was likely not significantly impacted by the distance of the trip [11].

Henry also had a history of gastrointestinal disturbances with the dish that he loved, and Henry of Huntingdon notes that lamprey “always disagreed with him” [6]. Henry’s prestigious court included some of the most renowned physicians and scholars of the twelfth century [1], and standard medical thought of the day considered lampreys a dangerous meal. Based on the humoral theories of the time, physicians thought that lampreys were “cold and moist in the fourth degree” and posed a “humoral threat” that could only be countered with spices such as black pepper “which is extremely hot and dry” [9]. They were also considered poisonous due to their resemblance to snakes [9]. Ironically, this made lamprey even more popular, in a manner comparable to modern consumption of the deadly Japanese blowfish [6].

There is precedent for lamprey poisoning in the modern day. Usually due to improperly removing the lamprey’s mucus covering and failure to properly wash the meat [8], subjects may experience poisoning symptoms several hours after ingestion, including nausea, vomiting, diarrhea, abdominal pain, and weakness [12]. Although the complete etiology is unknown, this disease is self-limited, and typically resolves within several days [8, 12]. However, as many researchers have noted, it is “disingenuous to suggest [that Henry I’s death] was a direct result of the meal itself” [7]. There are no reports of death via lamprey poisoning in modern literature [8]. Aside from a brief illness in late 1132, Henry enjoyed exemplary good health throughout his life, and his impressive level of productivity during 1135 gives “no indication that he might have been suffering from a serious ailment… nor can we suspect mental distress” [4]. He was also far from the only monarch to routinely ignore his doctors’ advice and overindulge in lampreys [4]. Given this, Henry of Huntingdon’s “theatrical description” appears highly suspect, particularly since other chroniclers of the time, including William of Malmesbury and Orderic Vitalis, make no mention of the lamprey meal [4]. Henry of Huntingdon also laid part of the blame on Henry’s daughter Matilda, claiming that the recent feud between the father and daughter provoked “angry and bitter feelings that contributed to the king’s demise” [4]. In this author’s opinion, the unlikely medical etiology of this casts doubt on Huntingdon’s reliability as a narrator.

Archbishop Hugh’s account of the king’s death also describes it as a “good death,” omitting any references to “unpleasant physical symptoms” [1]. While this may have been propaganda to immortalize the death of the monarch, it does raise the possibility that Henry did not experience the intense abdominal pain that would be expected of an etiology such as gastric obstruction or a perforated peptic ulcer, as some researchers have suggested [5].

A possible poisoning?

In late 1135, England stood upon the verge of a succession crisis. Although Henry had previously promised the throne to his daughter Matilda, his nephew Stephen had emerged as a serious contender for the crown. Henry and Matilda were feuding at the time of his death -- if she had managed to mend the divide before her father’s passing, Matilda may have been able to secure her position [2].

Stephen appears to have benefited significantly from his uncle’s sudden passing. While some nobles present at Henry’s deathbed swore that he had re-affirmed that the English throne should pass to his daughter, the cunning royal steward Hugh Bigod immediately “rushed across the Channel straight after the king’s death,” presented himself before the Archbishop of Canterbury, and played a pivotal role in “persuading the hesitant Archbishop to crown Stephen king on 22 December 1135” [2]. The speed with which Bigod accomplished this is remarkable; he succeeded in having Stephen crowned before the old king had even been buried [1].

Bigod emerges as a suspicious character in this story. A cunning politician, he lived to over the age of 80, served four different kings, and was a major political figure in European politics for most of the twelfth century [2]. While an important figure at court, he was largely seen as a “selfish man who held only lightly to his alliances… the only cause Hugh Bigod was ever interested in was that of Hugh Bigod” [2]. Henry’s sudden death, followed by Bigod’s near-instantaneous pivot to supporting Stephen, is suspicious. Henry had experienced multiple assassination attempts in the past. On one notable occasion in 1119, one of his daughters had tried to shoot him with a crossbow for allowing her two daughters to be blinded [1]. Poisoning, particularly with arsenic, was common in the Middle Ages [13] -- was Henry I’s sudden death due to poisoning by a close ally?

While it is possible, it is highly unlikely. Most of the assassination attempts against Henry occurred relatively early in his reign, with notable events during 1118, 1119, and 1121 [1]. By 1135, he was nearly universally beloved by his contemporaries, a “rex pacificus” seen as the “greatest monarch of his time” [14]. Henry had an uncanny ability to turn potential rivals into close allies and was exceptionally talented at developing “good personal relationships [as] a political art” [14], while simultaneously displaying the traits of a harsh and stern leader [1] -- in short, the ideal medieval king [14]. England was Europe’s “richest and most powerful regime” under his rule [2] -- a far cry from the depths to which the realm would soon descend in the bloody Anarchy [2].

Ultimately, there would have been little incentive for any of Henry’s nobles to assassinate him, nor were either of the claimants well-positioned at the time of his death. Both Stephen and Matilda were taken off-balance by his sudden passing, and it was only Stephen’s speed that let him lay claim to the crown first. In this context, Bigod’s support of Stephen’s claim [2] is far more likely due to have been due to simple political expedience rather than a dark conspiracy.

Other possible causes

If Henry’s death likely did not occur due to poisoning -- via lamprey or more nefarious means -- then the etiology remains in question. There is a possibility that his sudden illness may not have been gastrointestinal in nature. The chronicler William of Malmesbury claimed that Henry fell ill during the hunt of November 25th [4]. The cold November air may have indeed precluded Henry to an acute upper respiratory infection (URI) [15], leading to his later symptoms. However, an acute URI is unlikely -- typically the patient will experience warning symptoms for several days [15], and Henry’s instructions to his huntsmen to prepare for a hunt the following day strongly suggest that he was experiencing no ill symptoms by the conclusion of the hunt on November 25th [1]. None of his described symptoms -- chills, convulsions, and fever -- correlate with the expected sore throat, cough, shortness of breath, wheezing, and other symptoms that are typical of an acute URI [15].

Although he makes no mention of Henry’s lamprey dinner, the chronicler Orderic Vitalis agrees with Henry of Huntingdon that the king suddenly fell ill the night before a planned day of hunting [4]. The combination of the suddenness of the king’s illness and his symptoms suggests that he may have suffered a gastrointestinal ailment after all. While death via lamprey poisoning is almost completely unknown [8], more mundane food poisoning can be fatal in infants and the elderly [16]. Food poisoning was commonplace during the medieval period, especially given the “abhorrent” hygienic conditions involved in food preparation [17].

There are a number of common pathogens associated with acute food poisoning. Enterohemorrhagic Escherichia coli (EHEC) is associated with severe abdominal cramping, bloody diarrhea, and life-threatening hemolytic uremic syndrome (HUS), and may be transmitted by consuming contaminated products such as meat, milk, dairy products, or contaminated water [18]. However, none of the accounts of Henry’s death include descriptions of bloody diarrhea. Although this may have been an attempt to preserve the dignity of the dead monarch, it is unlikely. Henry of Huntingdon goes into extremely graphic detail describing the “strong, prevalent stench” and “fearful black fluid” that dripped from Henry I’s improperly preserved corpse [2], hardly a kingly image. In this author’s opinion, it is more likely that Henry simply did not produce bloody stool at all.

While over 200 food-borne diseases have been identified [19], the suddenness of Henry’s gastrointestinal symptoms suggests that he rapidly experienced a preformed toxin in his meal. *Bacillus cereus* and *Staphylococcus aureus* both cause a rapid onset of symptoms within several others due to the ingestion of preformed toxins [20]. While B. cereus is commonly known for its association with cooked rice dishes, it may be present in beef and poultry, and typically presents as a self-limited disease with significant abdominal cramping, nausea, and vomiting. However, deaths have been known to occur [20].

*Staphylococcus aureus* appears to be a more likely cause. The leading cause of foodborne intoxications in the modern world [21], this pathogen produces preformed enterotoxins that can cause acute food poisoning within 2-8 h [16]. In a castle in November, most of the foodstuffs would have likely been stored in unhygienic conditions [17], providing the optimal environment for *S. aureus* growth [16]. Symptoms may include nausea, vomiting, fever, and shivering [21]. While in most cases, the disease is self-limiting within approximately 24 h, fatalities have been known to occur [21]. Staphylococcal enterotoxins (SE) may cause violent vomiting, diarrhea, and fever. However, in severe cases, SEs may also cause toxic shock syndrome (TSS), due to their status as superantigens -- allowing them to bind to MHC-II and cause a massive overstimulation of T-cells [22]. However, even with this, the fatality rate for staphylococcal food poisoning is miniscule -- out of approximately 241,188 illnesses in the United States each year, only six will lead to death [19]. Though these modern statistics do not translate directly to the medieval world, it is unlikely that the hale and hearty Henry [4] would have died to such a contamination.

The most likely culprit is Listeria monocytogenes. This bacterium is foodborne, and as the causative agent of listeriosis [23], can have a mortality rate of up to 20%-30%, with complications that include meningitis, gastroenteritis, and septicemia [19]. In the modern day, it accounts for up to 27.6% of all foodborne disease fatalities [24]. The stone fortress of Lyons-la-Forêt [3] was likely cold and damp in November 1135, providing optimal conditions for Listeria growth [19]. The 67- or 68-year-old Henry also fits the demographic for Listeria infections, which are most commonly reported in those older than 64 [19]. Between 24% and 37% of patients with listeriosis have no pre-existing conditions [24].

A Gram-positive facultatively anerobic rod, Listeria has been isolated from cattle, sheep, goats, poultry, sewage, slaughterhouse waste, milk, human/animal feces [25], and undercooked meat [24] -- all factors that were ubiquitous in medieval settlements [17]. It will typically manifest sporadically in humans [25], as opposed to the sudden and large outbreaks associated with *S. aureus* food poisonings [21] -- perhaps explaining why none of Henry’s hunting companions are recorded as falling ill. It may manifest with central nervous system (CNS) infection, involving headache, vomiting, fever, and malaise [25] -- the very same malaise that may have convinced Henry that his death was imminent [2]. Other symptoms include septic arthritis, osteomyelitis, pleural infection, and cutaneous infections [24]. At the beginning stages of its gastrointestinal infection, it may incubate within 9-32 h [24], consistent with the timeline of Henry’s illness. As the disease progresses, meningitis becomes the most common form of listeriosis. Manifesting with “high fever, nuchal rigidity, movement disorders such as tremor and/or ataxia, and seizures” [26], the presentation appears similar to the historical descriptions of Henry’s “convulsions” [1]. Patients with CNS listeriosis are also less likely to have any sort of underlying medical condition than alternative forms of listeriosis infection [27], further supporting our theory that the healthy and fit Henry [1] experienced the neurological form of this disease. If he had had a meningitis secondary to listeriosis, it is likely that he experienced significant confusion and fatigue [28], perhaps explaining why the nobles at his bedside made wildly conflicting reports about who he designated as his successor [2]. The fatigue Henry would have experienced from this infection [28] may also explain why the Archbishop of Rouen regarded the king’s death as a “good death” without any significant pain [1].

However, the information currently available to the modern day remains frustratingly vague. Henry’s condition may have not been due to an acute episode of food poisoning -- other abdominal pathologies such as acute mesenteric ischemia, an abdominal aortic aneurysm, diverticulitis complicated by the development of sepsis, appendicitis, and various forms of biliary disease can all manifest as abdominal emergencies in older patients and lead to death if untreated [29]. Although Henry’s bowels were buried separately from his body [2], implying that there may have been an opportunity for an autopsy, human dissection was prohibited by the medieval Catholic Church [30]. Henry’s physicians would have been forbidden from performing any postmortem autopsy that would have indicated any possible structural components of his disease. Unfortunately, this is a key limitation of this article. While we are able to rule out the possibility of deliberate poisoning or poisoning via lamprey, the potential pathology of Henry’s disease is vast, and the evidence remarkably limited.

It is difficult to make a conclusive diagnosis based on the vagueness of Henry’s symptoms. However, the suddenness with which the disease struck him -- a fact emphasized by all of the chroniclers [1] -- and the fever, convulsions, malaise, and possible confusion that he experienced [1] lead us to suggest that it was likely a sporadic case of Listeria monocytogenes that primarily manifested as a CNS infection. While Henry of Huntingdon’s description of the king’s death due to an overindulgence of lampreys makes for a “theatrical” tale that was later widely adopted by historians [4], the rare and self-resolving cases of lamprey poisoning documented in the literature do not mesh with the king’s symptoms. Instead of dying of greed or gluttony, as some historians have implied [5], Henry may have been unlucky enough to become infected with one of the deadliest foodborne pathogens in the world [24]. While it is impossibly to conclusively determine the full etiology of Henry’s disease, both a deliberate assassination and death via a “surfeit of lampreys” [7] can be safely discounted as plausible theories.

## Conclusions

While the description of Henry I’s death due to overeating a large meal of lampreys makes for an interesting historical anecdote, the tale does not appear to have any firm basis in reality. While it is impossible to conclusively determine the cause of Henry’s sudden illness and death, we conclude that the possibility of his death due to assassination or to lamprey poisoning is highly unlikely. If the king suffered from food poisoning, we believe that an acute meningitis secondary to Listeria monocytogenes is the most likely culprit for the king’s strange symptoms and sudden passing. However, the wide variety of other potentially lethal abdominal pathologies, including pancreatitis, appendicitis, biliary disease, should also be considered in this interesting historical case.

## References

[1] Green JA (2006). Henry I: King of England and Duke of Normandy.

[2] Huscroft R (2016). Tales from the Long Twelfth Century: The Rise and Fall of the Angevin Empire.

[3] Lepeuple B (2008). Lyons-la-Forêt (Eure). Château. Mediev Archaeol.

[4] Hollister CW (2001). Henry I.

[5] Davison MA (1964). Medicine, murder, and man. Med Leg J.

[6] Freedman P (2021). Lamprey and herring. Imago Temporis - Medium Aev.

[7] Docker MF, Hume JB, Clemens BJ (2015). Introduction: a surfeit of lampreys. Lampreys: Biology, Conservation and Control: Volume 1.

[8] Hanel L, Andreska J, Dyldin YV (2022). Lampreys in human life, their cultural and folklore importance. Human Soc Sci.

[9] Freedman P (2021). Luxury dining in the Middle Ages. Ex-poisition, National Taiwan University.

[10] Adamson MW (2004). Food in Medieval Times.

[11] Renaud CB (2011). Lampreys of the world: an annotated and illustrated catalogue of Lamprey species known to date. https://www.researchgate.net/publication/264201079_Lampreys_of_the_World_An_Annotated_and_Illustrated_Catalogue_of_Lamprey_Species_Known_to_Date.

[12] Halstead BW (1967). Poisonous and Venomous Marine Animals of the World.

[13] Nriagu JO (2002). Arsenic poisoning through the ages. Environmental Chemistry of Arsenic.

[14] Hollister CW (1976). The taming of a turbulent Earl: Henry I and William of Warenne. Hist Reflect.

[15] Mourtzoukou EG, Falagas ME (2007). Exposure to cold and respiratory tract infections. Int J Tuberc Lung Dis.

[16] Argudín MÁ, Mendoza MC, Rodicio MR (2010). Food poisoning and Staphylococcus aureus enterotoxins. Toxins (Basel).

[17] Satin M (2009). Death in the Pot: The Impact of Food Poisoning on History.

[18] Nguyen Y, Sperandio V (2012). Enterohemorrhagic E. coli (EHEC) pathogenesis. Front Cell Infect Microbiol.

[19] Bintsis T (2017). Foodborne pathogens. AIMS Microbiol.

[20] Schoeni JL, Wong AC (2005). Bacillus cereus food poisoning and its toxins. J Food Prot.

[21] Fetsch A, Johler S (2018). Staphylococcus aureus as a foodborne pathogen. Curr Clin Microbiol Rep.

[22] Etter D, Schelin J, Schuppler M, Johler S (2020). Staphylococcal enterotoxin C - an update on SEC variants, their structure and properties, and their role in foodborne intoxications. Toxins (Basel).

[23] Gandhi M, Chikindas ML (2007). Listeria: a foodborne pathogen that knows how to survive. Int J Food Microbiol.

[24] Painter J, Slutsker L (2007). Listeriosis in humans. Listeria, Listeriosis, and Food Safety: Third Edition.

[25] Farber JM, Peterkin PI (1991). Listeria monocytogenes, a food-borne pathogen. Microbiol Rev.

[26] Doganay M (2003). Listeriosis: clinical presentation. FEMS Microbiol Immunol.

[27] Gerner-Smidt P, Ethelberg S, Schiellerup P (2005). Invasive listeriosis in Denmark 1994-2003: a review of 299 cases with special emphasis on risk factors for mortality. Clin Microbiol Infect.

[28] Norton DM, Braden CR (2007). Foodborne listeriosis. Listeria, Listeriosis, and Food Safety: Third Edition.

[29] Spangler R, Van Pham T, Khoujah D, Martinez JP (2014). Abdominal emergencies in the geriatric patient. Int J Emerg Med.

[30] Bradley EL 3rd, Dexter ND (2010). Management of severe acute pancreatitis: a surgical odyssey. Ann Surg.

